# Draft Genome Sequence of a Multidrug-Resistant Strain of Salmonella enterica Serovar Typhimurium Isolated from a Pine Siskin (Spinus pinus)

**DOI:** 10.1128/mra.00982-22

**Published:** 2022-10-10

**Authors:** Tricia A. Van Laar, Lindsey Biehler, Joel W. G. Slade

**Affiliations:** a Department of Biological Sciences, California State University, Stanislaus, Turlock, California, USA; b Department of Biology, California State University, Fresno, Fresno, California, USA; University of Maryland School of Medicine

## Abstract

A recent outbreak of salmonellosis in wild birds sickened 29 individuals in 12 states, leading to 14 hospitalizations. Here, we report the draft genome sequence of a multidrug-resistant strain of Salmonella enterica serovar Typhimurium that was isolated from a bird experiencing symptoms of salmonellosis.

## ANNOUNCEMENT

Feeding birds is a common hobby for those who live in cities and rural areas (1). Bird feeders can help granivorous birds gain supplemental nutrition, but the lack of sanitary conditions at bird feeders often results in them becoming rich with microorganisms (2). One disease that can spread at bird feeders is salmonellosis caused by Salmonella enterica serovar Typhimurium (3). From December 2020 to May 2021, an avian Salmonella outbreak on the west coast of the United States caused some people who handled bird feeders and lethargic songbirds to contract the pathogen and experience symptoms of human salmonellosis (4). In February 2021, a pine siskin (Spinus pinus) was discovered in Fresno, California, with symptoms of salmonellosis and succumbed to this disease.

A sterile, premoistened (10 mM Tris, 1 mM EDTA, 0.05% [vol/vol] Tween 20 [pH 8.0]) cotton swab was used to sample the cloaca of the bird. The sample was swabbed on Salmonella*-Shigella* agar (BD Difco) to selectively isolate putative Salmonella colonies. A single black colony (PS01) was grown overnight in Luria-Bertani broth. Whole-genomic DNA was extracted using the Wizard genomic DNA purification kit (Promega, Madison, WI).

Library preparation using the Illumina DNA preparation kit and TDT 10-bp unique dual indexing (UDI) indices and whole-genome sequencing using an Illumina NextSeq 2000 system were provided by the Microbial Genome Sequencing Center (MiGS) (Pittsburg, PA), producing 2 × 151-bp reads (3,757,134 total reads [average read length, 147 bp]). Demultiplexing, quality control, and adapter trimming were performed with BCL Convert v3.9.3 (Illumina). Assembly was performed using the St. Petersburg Genome Assembler (SPAdes v3.15.5 [5]), and quality metrics were determined using QUAST v5.2 (6). The draft genome is 4,802,034 bp, with a G+C content of 52.13%. There are 111 contigs, with a contig *N*_50_ value of 149,367 bp. We calculated the average nucleotide identity (ANI) with fastANI v1.33 (7) and found similarity to *S*. Typhimurium 138736 at 99.87% and to S. enterica serovar Typhi 404ty at 96.14%. We used the NCBI Prokaryotic Genome Annotation Pipeline (PGAP) v6.2 (8) to annotate the genome and found 4,499 coding sequences and 97 RNAs. Default parameters were used for all software.

Because the 2020–2021 *S*. Typhimurium outbreak in wild birds was associated with human illness, we wanted to determine whether there was antimicrobial resistance in this isolate. We used the Comprehensive Antibiotic Resistance Database (CARD) (9) to predict the presence of cassettes conferring resistance to numerous classes of antibiotics, including β-lactams, aminoglycosides, fluoroquinolones, and tetracyclines.

The emergence of zoonotic, antimicrobial-resistant bacteria in birds that frequent feeders presents a concern for hobbyist birders. The data from this study can be used to inform birders to keep feeders clean, remove feeders during times of disease outbreak, and thoroughly wash their hands after handling objects that might have come into contact with wild birds.

### Data availability.

This whole-genome shotgun project has been deposited in DDBJ/ENA/GenBank under the accession number JANWGZ000000000. The version described in this paper is version JANWGZ010000000. The raw sequences have been deposited in the NCBI SRA under the accession number SRR21019529.
